# Sociodemographic and health-related factors associated with viral load non-suppression and body mass index in adults with depression symptoms receiving antiretroviral therapy in South Africa

**DOI:** 10.1371/journal.pone.0329990

**Published:** 2026-02-23

**Authors:** Babalwa Zani, Lara Fairall, Inge Petersen, Naomi Folb, Arvin Bhana, Graham Thornicroft, Jill Hanass-Hancock, Oné Selohilwe, Ruwayda Petrus, Sergio Carmona, Carl Lombard, Crick Lund, Naomi Levitt, Max Bachmann

**Affiliations:** 1 Knowledge Translation Unit, Department of Medicine, Faculty of Health Sciences, University of Cape Town, Cape Town, South Africa; 2 King’s Global Health Institute, School of Life Course & Population Sciences, Faculty of Life Sciences and Medicine, King’s College London, London, United Kingdom; 3 Centre for Rural Health, School of Nursing and Public Health, University of KwaZulu-Natal, Durban, South Africa; 4 Health Systems Research Unit, South African Medical Research Council, Durban, South Africa; 5 Centre for Global Mental Health, and Centre for Implementation Science, Health Service and Population Research Department, Institute of Psychiatry, Psychology and Neuroscience, King′s College London, London, United Kingdom; 6 Gender and Health Research Unit, South African Medical Research Council, Durban, South Africa; 7 School of Health Sciences, University of KwaZulu-Natal, Durban, South Africa; 8 Department of Molecular Medicine and Haematology, University of the Witwatersrand, National Health Laboratory Service, Johannesburg, South Africa; 9 Biostatistics Unit, South African Medical Research Council, Cape Town, South Africa; 10 Division of Epidemiology and Biostatistics, Department of Global Health, University of Stellenbosch, Cape Town, South Africa; 11 Alan J Flisher Centre for Public Mental Health, Department of Psychiatry and Mental Health, University of Cape Town, Cape Town, South Africa; 12 Chronic Disease Initiative for Africa, Department of Medicine, Faculty of Health Sciences, University of Cape Town, Cape Town, South Africa; 13 Norwich Medical School, University of East Anglia, Norwich, United Kingdom; Public Library of Science, UNITED KINGDOM OF GREAT BRITAIN AND NORTHERN IRELAND

## Abstract

**Introduction:**

While antiretroviral therapy (ART) has significantly improved HIV outcomes, viral load remains unsuppressed for 6% of the people on ART globally in 2024. In South Africa, 5.7 million people are on ART, and viral load non-suppression was reported in 8% of them in 2022. Viral load non-suppression during ART is associated with health decline and HIV transmission. Weight is also a vital component for the management of HIV. High body mass index (BMI) increases the risk of non-communicable diseases, increasing the risk of multimorbidity in people living with HIV. Both ART effectiveness and obesity have been shown to be affected by socioeconomic, psychological and health related factors, but their interrelationships in South Africans living with HIV are not well known. This study aims to investigate the effects of socioeconomic and health related factors at enrolment, and their changes over time in viral load non-suppression and BMI among people receiving ART who have depression symptoms.

**Methods:**

This was a secondary analysis of data from a randomised controlled trial of depression management in 2002 adults receiving ART. We investigated the effects of sociodemographic characteristics, comorbidities, depression symptoms (Patient Health Questionnaire-9 (PHQ-9)), functional disability (WHODAS-2.0), AIDS-related stigma and ART adherence – all measured at baseline – on viral load non-suppression (viral load ≥1000 copies/ml) and on body mass index (BMI), at baseline and on changes 12 months later, using longitudinal mixed effect logistic and linear regression models. A P-value of 0.05 or less was considered statistically significant. Potentially confounding covariates were selected and adjusted for using least absolute shrinkage and selection operator (LASSO) inference as a sensitivity analysis.

**Results:**

People with viral load non-suppression at baseline were more likely to be male, younger and to earn lower income. Health characteristics associated with viral load non-suppression at baseline were previous tuberculosis, having been on ART for less than 6 months or more than 10 years, and self-reported non-adherence to ART. Higher disability score and ART duration <6 months or >10 years at baseline were associated with an increasing likelihood of viral load non-suppression 12 months later. Higher BMI at baseline was associated with being female, being married, earning higher income and hypertension, no history of tuberculosis and not having viral load non-suppression. BMI increased from baseline to follow-up, and women and younger people had greater increases in BMI 12 months later. Depression symptom scores and stigma scores were not associated with viral load non-suppression or BMI.

**Conclusions:**

This study identified sociodemographic risk factors associated with viral non-suppression in PLWH, but most of them were not associated with further changes over time. Functional disability, however, was a risk factor with long-term implications. Younger people and women were at greater risk of BMI increasing over time. This suggests a need for ART programs to integrate long-term support services like frequent adherence assessment, mental health, rehabilitation and weight management strategies tailored to high-risk groups.

ClinicalTrials.gov (NCT02407691), Pan African Clinical Trials Registry (201504001078347), South African National Clinical Trials Register (SANCTR) (DOH-27-0515-5048, NHREC 4048).

## Introduction

HIV continues to be a serious global public health concern with 40.8 million people living with HIV in 2024 [1]. While antiretroviral therapy (ART) has significantly improved HIV outcomes, viral load remains unsuppressed for 6% of the people on ART globally in 2024 [1]. South Africa delivers the largest ART programme globally, with over 5.7 million people receiving ART in 2022. The Joint United Nations Programme on HIV/AIDS (UNAIDS) estimated the prevalence of viral load suppression (HIV RNA < 1000 copies/mL) to be at 92% in the treated population in South Africa in 2022 [2]. Viral load suppression rates in the treated population vary between provinces and districts. In the North West Province in 2015, at the time of this study, viral load suppression was estimated at 74% [3]. Viral load suppression is critical in HIV care as continued viral load non-suppression is associated with increased disease progression, health decline, acquisition of resistance to medication, higher risk of HIV transmission and death [4,5]. Even in resource-constrained settings, viral load monitoring is key to achieving desired targets and identification of high-risk people for targeted interventions and factors which may compromise suppression need to be monitored.

Systematic reviews have found that males, younger people and people who have lower household income were more likely to have viral load non-suppression [6,7]. Health characteristics associated with viral load non-suppression included sub-optimal adherence to ART, depression, having been on ART for a shorter duration, reporting a history of TB and having a higher BMI [6–8]. These studies reported the effect on viral load non-suppression of age, depression, duration on ART and history of TB among children and adolescents [9–14]. Most of these studies were cross-sectional, limiting inferences about direction of effect. Gaps also remain in the effects of ART history, health characteristics and disability on viral load non-suppression.

As ART programmes mature, PLWH are living longer and developing comorbidities, including cardiovascular and metabolic conditions. This can be attributed in part to the rising prevalence of overweight and obesity among PLWH. Obesity and higher BMI are increasing among PLWH as in the general population [15,16]. Although weight gain after ART initiation may reduce mortality risk in underweight individuals, overweight and obesity increase the risk for high blood pressure as well as metabolic and cardiovascular disease [15,17]. The prevalence of obesity is greater in Black women and in people of low socioeconomic status [15]. Excess caloric intake, high-fat diets, and reduced physical activity promote weight gain and obesity both among PLWH and in the general population [18,19]. Studies from mostly the USA and Switzerland have shown high viral load to be associated with greater weight gain after ART initiation [20–22]. In South Africa, obesity among pregnant and post-partum women is more common with viral load non-suppression [23], but this relationship is unclear among the general population of PLWH.

We hypothesized that depression symptoms, stigma and disability would lead to lower adherence, which would lead to viral load non-suppression and worse clinical outcomes including weight loss. Comorbidities could also lead to increased depression symptoms and disability, with similar consequences for adherence and ART outcomes. We also hypothesized that viral load non-suppression, and adverse socioeconomic, psychological and physical health conditions, could be associated with BMI.

The aim of this study was to investigate the effects of depression symptom severity, stigma, disability, comorbidity and socioeconomic factors on viral load non-suppression and on BMI in people receiving ART who have depression symptoms. The specific objectives were to investigate whether these factors, measured at baseline, were associated with i) viral load non-suppression and BMI measured at baseline and ii) changes in viral load non-suppression and BMI from baseline to 12-month follow-up.

## Materials and methods

### Study design and population

This study was a secondary analysis of data collected from participants enrolled in the Comorbid Affective Disorders, AIDS/HIV, and Long Term Health (CobALT) trial [24]. While the primary study used randomization to allocate participants to intervention and control arms, this secondary analysis study used data collected from participants in both arms combined. Data were collected at baseline and after 12 months follow-up. We investigated associations between two physical health outcomes – HIV viral load non-suppression and BMI – measured at baseline and at 12 months, and depression symptom severity, stigma, disability, comorbidity and socioeconomic factors measured at baseline.

CobALT was a pragmatic, two-arm cluster randomised trial conducted in 40 largest clinics in the Dr Kenneth Kaunda and Bojanala districts, North West province of South Africa. Participants were recruited from 21 April 2015–14 December 2016 [25] and follow-up interviews and data collection were conducted up to 18 January 2018. The trial assessed the effectiveness of a collaborative care model for the detection and management of depression on viral load non-suppression and depression symptoms among people on ART attending primary care facilities. Eligible participants were 18 years or older, on ART, and screened positive for depression symptoms as indicated by a score of nine or more on the PHQ-9, which was the established cut-off for depression in people with chronic conditions in the North West Province [26]. The trial showed that the intervention had no effects on depression symptoms or viral load non-suppression (the trial’s primary outcomes) or on any secondary outcomes including BMI [24].

### Data collection and management

We collected data using questionnaires administered by trained interviewers at baseline and at 12 months, separate from clinical care. Depression symptoms were assessed using a locally translated (Setswana) and validated version of the PHQ-9, with an inclusion cut-off score of nine [26,27]. The PHQ-9 is a nine-item measure corresponding to the criteria upon which a diagnosis of depressive disorders is based in the Diagnostic and Statistical Manual of Mental Disorders (DSM-IV). Items are scored based on frequency of responses ranging from 0 (not at all) to 3 (nearly every day) and summed assuming equal weighting to yield a total score from 0 to 27, with higher scores indicating more severe depression symptoms.

The baseline questionnaire covered demographic details, level of education, employment status, income and self-reported comorbidities. Comorbidities were reported if a diagnosis had ever been given by a health care worker of stroke, heart attack or angina (ischaemic heart disease (IHD)), hypertension or tuberculosis (TB). The questionnaire included several standardised scales including the Internalised AIDS-Related Stigma Scale [28], the Perceived Stress Scale [29] and the 12-item World Health Organization Disability Assessment Schedule (WHODAS-2.0), which measures functional limitations [30–33]. We also recorded participants’ history on ART, including the date they initiated ART, the medication they were taking and the most recent viral load results. These interview data were complemented by examination of participants’ clinic folders. We used a visual analogue scale (VAS) ranging from 0–100% to measure self-reported adherence to ART in the last month as reported to trained fieldworkers separate from clinical care. Values below 90% were categorised as non-adherent. The cut-off was derived from a prior validation study that demonstrated a strong correlation in adherence rates and yielded consistent classification of optimal (>90%) and suboptimal (<90%) adherence between VAS and established instruments [34]. Duration on ART was calculated as the number of months between the time the participant-initiated ART and the date of their baseline interview.

We measured weight using a digital scale and height using a wall mounted height meter and used these to calculate BMI, defined as the body mass divided by the square of the body height (kg/m^2^). We categorised ART duration as < 6 months, 6–120 months, and > 120 months. Longer duration on ART was categorised as having been on ART for 10 years (120 months) or more, as a previous study in South Africa showed an increase in the probability of virological failure and of switching to second-line therapy after being on ART for 10 years [35].

Viral load was assessed at baseline and at 12 months. At baseline in the Dr Kenneth Kaunda district, we recorded the most recent available viral load result from clinic files. For study participants with no viral load in the preceding six months, we collected blood samples for viral load analysis. Follow-up viral loads were assessed for all participants. In the Bojanala district, we collected blood samples for all participants at baseline and follow-up. We also obtained routine viral load results from the National Laboratory Health Services (NHLS). Blood samples for research-funded viral load analysis were collected at the clinics using standard procedures and processed with routine blood samples at the NHLS.

We defined viral load non-suppression as HIV RNA ≥ 1000 copies/mL [36]. Participants with missing viral load results were excluded in the analysis.

### Statistical analysis

First, we investigated crude associations between participants’ depression symptom severity, stigma and disability scores, comorbidities, socioeconomic factors (the explanatory or independent variables) and viral load non-suppression and BMI (outcome variables) measured at baseline and 12-month follow-up. PHQ-9 score, stigma score and disability score were standardised by subtracting the mean from each measurement and dividing by the standard deviation for use as regression covariates. P-values were estimated for each explanatory variable separately, using logistic regression models (with viral load non-suppression as outcome) or linear regression models (with BMI as outcome), with robust adjustment for clustering of outcomes within clinics [37,38]. To compare the magnitude of differences between participants with and without detectable viral loads among variables measured with different scales, we calculated standardized differences in baseline variables using Cohen’s formulae for differences in means and proportions [39].

Secondly, we investigated the mutually adjusted associations between the explanatory variables, measured at baseline, and the viral load non-suppression and BMI outcomes measured at baseline and 12-month follow-up, using multilevel longitudinal mixed effects regression models [40]. A linear model was used for BMI as a continuous outcome and a generalized linear model with a logit link was used for viral load non-suppression as binary outcome. Individuals and clinics were modelled as having random intercepts, with individuals nested within clinics, to account for repeated measurement of outcomes in individuals, and randomisation of clinics. Individuals were random effects. The explanatory variables of interest, recorded at baseline, were sociodemographic indicators (age, sex, marital status, household income); depression symptoms (PHQ-9), stigma and disability scores; comorbid conditions (hypertension, IHD, previous TB) and ART history (non-adherence, ART started less than 6 months or more than 10 years ago). These variables were fixed effects. For models with BMI as an outcome we used viral load non-suppression instead of ART history as an explanatory variable because we assumed that effects of ART history on BMI would be mediated through viral load non-suppression (Supplementary Figure). All models included trial arm (intervention or control) as a covariate to account for the origin of the data in a randomised trial, although the purpose of present study was not to estimate the effects of the intervention.

To account for the longitudinal study design, data were arranged so that each participant had two records. The first record comprised baseline measures of the explanatory variables, the baseline measures of viral load non-suppression and BMI, trial arm, and a dummy time variable coded as 0. The second record comprised the same baseline measures of the explanatory variables, the 12-month follow-up measures of viral load non-suppression and BMI, and a dummy time variable coded as 1. To estimate the effects of each baseline explanatory variable on changes in outcomes we included covariate-time interaction variables. The coefficients for these interaction variables quantified the estimated effect of the baseline explanatory variables on changes in the outcome from baseline to 12-month follow-up. The longitudinal mixed effect models were the primary analysis.

Thirdly, as a sensitivity analysis to investigate whether these models may have missed possible statistically significant associations between outcomes and explanatory variables because of over-adjustment for numerous correlated covariates, we constructed more parsimonious regression models using LASSO (least absolute shrinkage and selection operator) inference methods [41,42]. For each explanatory variable of interest, LASSO algorithms select a subset of the covariates that are associated either with the outcome of interest, or with the explanatory variable of interest, or both. Covariate selection is based on the magnitude of their coefficients and not on p-values. The selected variables are then included in a final linear or logistic regression model which estimates the adjusted association between the explanatory variable of interest and the outcome. We used Stata’s double selection inference applications ‘dsregress’ and ‘dslogit’ [43]. Because these applications do not support multilevel mixed models, we used separate models for outcomes measured at baseline and at follow-up, with variance correction for clustering of outcomes within clinics. Models with each 12-month follow-up value as outcome included baseline values of the same variable as a covariate; the model coefficients therefore represent associations between the explanatory variables and changes in the outcome [44].

Statistical analyses were performed with Stata version 18 statistical software [45]. A P-value of 0.05 or less was considered statistically significant.

### Ethical considerations and trial registration

The trial was registered with ClinicalTrials.gov (NCT02407691), the Pan African Clinical Trials Registry (201504001078347) and the South African National Clinical Trials Register (DOH-27-0515-5048NHREC number 4048). Ethical approval for the trial was obtained from the University of Cape Town Human Research Ethics Committee (211/2013), King’s College London Research Ethics Office (PNM/12/13–159) and the University of KwaZulu-Natal Biomedical Research Ethics Committee (BFC049/15). Permission to conduct the study was granted by the North West Provincial Department of Health. All patient participants provided written informed consent to participate in the study. The study was monitored by a Data and Safety Monitoring Board (DSMB), holding meetings twice a year for the duration of the study.

## Results

A total of 2002 participants were enrolled and interviewed at baseline, 1008 in the intervention and 994 in the control arm; of whom 1675 (84%) were interviewed again at 12 months. Of the enrolled participants, 1876 (94%) had viral load results available at baseline and 1837 (92%) at 12 months. Socioeconomic and health characteristics of the participants are shown in Table 1. Most participants were black, women and unmarried, and half were aged 42 years or older. The average personal monthly income was low: equivalent to US$ 72.05 in 2016 (ZAR 1060) [46]. The mean PHQ-9 score at baseline was 14, with 65% of participants having a score of 9–14 corresponding to mild depression symptoms.

**Table 1:** Socioeconomic, psychological and health characteristics of participants with or without viral load suppression at baseline and at 12 months.

Characteristics	At baseline					At 12 months				
	All participants^1^ n (%)	HIV RNA ≥1000 copies/ml: n (%)	HIV RNA <1000 copies/ml: n (%)	sMD	p-value*	All participants^1^ n (%)	HIV RNA ≥1000 copies/ml: n (%)	HIV RNA <1000 copies/ml: n (%)	sMD	p-value*
**Total participants**	**N = 1876**	**1574 (86%)**	**302 (14%)**			**N = 1837**	**1585 (85%)**	**282 (15%)**		
**Socioeconomic characteristics**										
Age (years): mean (SD)	42(11)	40 (10)	42 (11)	**0.20**	**0.004**	42 (11)	40 (11)	43 (11)	**0.23**	**0.002**
Male	340 (18)	78 (23)	262 (77)	**0.23**	**<0.001**	320 (17)	65 (20)	255 (80)	**0.17**	**0.023**
Female	1536 (82)	224 (15)	1312 (85)			1517 (83)	217 (14)	1300 (86)		
Married	437 (23)	55 (13)	382 (87)	**0.15**	**0.034**	415 (23)	51 (12)	364 (88)	0.13	0.067
Unmarried	1439 (77)	247 (17)	1192 (83)			1422 (77)	231 (16)	1191 (84)		
Total income ^2, 3^	1.1 (1.7)	0.8 (1.5)	1.1 (1.8)	**0.15**	**0.042**		0.9 (1.63)	1.1 (1.8)	0.11	0.172
**Psychological characteristics, disability and BMI**										
PHQ-score: mean (SD)	13.7 (4.0)	14.2 (4.1)	13.6 (4.0)	**−0.13**	**0.023**	13.7 (4.0)	13.9 (4.2)	13.7 (4.0)	−0.05	0.431
Stress score: mean (SD)	20.8 (6.6)	20.9 (6.2)	20.7 (6.7)	−0.02	0.770	20.7 (6.6)	21.1 (6.3)	20.6 (20.3)	−0.07	0.319
Stigma score: mean (SD)	2.7 (2.1)	2.5 (2.1)	2.8 (2.1)	0.12	0.082	2.7 (2.1)	2.5 (2.3)	2.7 (2.6)	0.11	0.098
Disability score: mean (SD)	10.4 (7.7)	10.7 (7.5)	10.3 (7.9)	−0.06	0.345	10.3 (7.7)	11.2 (7.6)	10.1 (7.7)	−0.14	0.053
BMI	N = 1854					N = 1812				
BMI: Mean (SD)	26.5 (7.9)	22.4 (5.5)	25.0 (6.8)	**0.38**	**<0.001**	25.0 (6.7)	22.9 (5.8)	25.0 (6.8)	**0.31**	**0.008**
**ART experience**										
Self-reported adherence <90% at baseline	211 (11)	67 (32)	144 (68)	**0.36**	**<0.001**	193 (11)	59 (31)	134 (69)	**0.35**	**<0.001**
Self-reported adherence ≥90% at baseline	1665 (89)	235 (14)	1430 (86)			1644 (89)	223 (14)	1421 (86)		
ART duration 6 months to 10 years	1347/1614 (83)	167 (12)	1180 (88)	**0.32**	**<0.001**	1290/1564 (82)	168 (13)	1122 (87)	**0.14**	**0.028**
ART duration < 6 months or > 10 years	267/1614 (17)	65 (24)	202 (76)			274/1564 (18)	52 (19)	222 (81)		
Viral load non-suppression at baseline						274/1724 (16)	160 (58)	114 (42)	**−1.72**	**<0.001**
Viral load suppression at baseline						1450/1724 (84)	101 (7)	1349 (93)		
**Self-reported comorbidity**										
Hypertension diagnosis or treatment	591 (32)	90 (15)	501 (85)	0.04	0.461	589 (32)	79 (13)	510 (87)	0.10	0.181
No hypertension diagnosis or treatment	1285 (68)	212 (16)	1073 (84)			1248 (68)	203 (16)	1045 (84)		
Ischaemic heart disease	404/1874 (22)	82 (20)	322 (80)	**0.05**	**0.009**	411	68 (17)	343 (83)	0.03	0.446
No ischaemic heart disease	1470/1874 (78)	220 (15)	1250 (85)			1423	214 (15)	1209 (845)		
Previous tuberculosis diagnosis	660/1874 (35)	147 (22)	513 (78)	**0.33**	**<0.001**	659/1834 (36)	129 (20)	530 (80)	**0.24**	**<0.001**
No previous tuberculosis diagnosis	1214/1874 (65)	155 (13)	1059 (87)			1175/1834 (64)	153 (13)	1022 (87)		

^1^The denominator for variables that have missing responses are indicated individually; for other variables the denominators are total participants.^2^Total income is total grant, employment and business income per person. ^3^1000 South African Rand per month per person. sMD = Standardised mean difference. SD = standard deviation. *P-values from logistic regression models with robust adjustment for intra-clinic correlation of outcomes

Of 1876 participants with viral load results, 1574 (84%) had viral load suppression at baseline and 1585 (85%) at 12 months (Table 1). At baseline, 11% of participants reported ART adherence of less than 90%. The median duration on ART was 37.9 months (interquartile range (IQR) 14.9–65.7 months); 12% had been on ART for less than 6 months and 5% for more than 120 months.

### Factors associated with viral load non-suppression at baseline and 12 months

In the bivariable analysis (Table 1), viral load non-suppression at baseline was significantly more common in men, in participants who were younger, unmarried, and those who earn a lower income. Participants with viral load non-suppression had higher mean scores for depressive symptoms, lower mean BMI, more frequently reported previous IHD, previous TB diagnosis, lower levels of ART adherence and having been on ART for less than 6 months or more than 120 months. Unsuppressed viral load at 12 months followed similar patterns, however, depressive symptoms, income and IHD no longer showed a significant difference (Table 1). Standardized differences between participants with and without viral load suppression were greatest for BMI, nonadherence to ART, tuberculosis and duration of ART at baseline and for BMI and nonadherence to ART at 12 months.

In the mixed effects logistic regression model (Table 2), socioeconomic characteristics associated with viral load non-suppression at baseline were younger age, being male, and earning a lower income. Health characteristics associated with viral load non-suppression were self-reported non-adherence to ART, having been on ART for less than 6 months or more than 10 years (120 months) and reporting a history of tuberculosis. Higher disability score at baseline was associated with increasing likelihood of having viral load non-suppression from baseline to follow-up and having been on ART for less than 6 months or more than 10 years at baseline was associated with deceasing likelihood of viral load non-suppression from baseline to follow-up. The LASSO models showed similar associations, except that the association between ART duration less than 6 months or more than 10 years was not associated with reduced odds of having viral load non-suppression at 12 months (Table 3).

**Table 2:** Sociodemographic and health characteristics measured at baseline and their association with viral load non-suppression at baseline, and with changes in viral load non-suppression from baseline to 12 months: longitudinal logistic regression mixed model including all covariates.

Characteristics	Outcome: Viral load non-suppression (HIV RNA ≥ 1000 copies/ml)		
	OR	95% CI	p-value
**Outcome: Viral load non-suppression (HIV RNA ≥ 1000 copies/ml) at baseline**			
**Socioeconomic characteristics**			
Age (per year)	0.96	0.93; 0.99	**0.005**
Female	0.29	0.14; 0.61	**0.001**
Married	0.58	0.27; 1.20	0.142
Total income (per ZAR 1000/month)	0.80	0.64; 0.98	**0.032**
**Psychological and health characteristics**			
PHQ-9 score (per standard deviation)	1.16	0.85; 1.59	0.341
Stigma score (per standard deviation)	0.91	0.68; 1.22	0.519
Disability score (per standard deviation)	1.05	0.76; 1.45	0.779
Ischaemic heart disease	1.69	0.72; 3.97	0.224
Hypertension diagnosis	1.23	0.63; 2.41	0.551
History of TB	3.01	1.61; 5.65	**0.001**
**ART experience**			
Self-reported adherence ≥90%	0.10	0.04; 0.23	**<0.001**
ART duration <6 months or >120 months. vs 6–120 months	8.17	3.57; 18.70	**<0.001**
**Trial design**			
Intervention vs. control arm	0.90	0.41; 1.99	0.788
**Outcome: Change in viral load non-suppression (HIV RNA ≥ 1000 copies/ml) at 12 months** ^**1**^			
**Socioeconomic characteristics**			
12-months vs. baseline	0.74	0.14; 3.66	0.699
Age	1.01	0.98; 1.04	0.585
Female	1.44	0.68; 3.05	0.347
Marriage	1.23	0.57; 2.65	0.596
Income	1.04	0.84; 1.28	0.751
**Psychological and health characteristics**			
PHQ-9 score (per standard deviation)	0.83	0.61; 1.13	0.234
Stigma score (per standard deviation)	0.89	0.66; 1.20	0.459
Disability score (per standard deviation)	1.50	1.08; 2.08	**0.017**
Ischaemic heart disease	0.62	0.26; 1.47	0.276
Hypertension diagnosis	0.62	0.31; 1.24	0.173
History of TB	0.62	0.33; 1.16	0.131
**ART experience**			
Self-reported adherence ≥90%	1.32	0.58; 2.98	0.503
ART duration <6 months or >120 months. vs 6–120 months	0.30	0.13; 0.69	**0.004**
**Trial design**			
Intervention vs. control arm	0.79	0.44; 1.43	0.443

^1^ORs for change in outcome estimated with covariate-time interaction terms. OR=Odds Ratio, CI = Confidence Interval.

**Table 3:** Sociodemographic and health characteristics measured at baseline associated with viral load non-suppression at baseline and with changes in viral load non-suppression from baseline to 12 months: logistic regression models with odds ratio for each variable adjusted for potentially confounding covariates selected using LASSO.

Outcome and explanatory variables	Viral load non-suppression (HIV RNA ≥ 1000 copies/ml)			
	OR	95% CI	P-value	selected control variables ^2,3^
**Outcome: Viral load non-suppression (HIV RNA ≥ 1000 copies/ml) at baseline** ^**1**^				
**Socioeconomic characteristics**				
Age (per year)	0.98	0.96; 0.99	**0.002**	sex, hypertension, ischaemic heart disease,
Female	0.63	0.44; 0.90	**0.011**	age
Married	0.74	0.51; 1.08	0.119	–
Total income (per ZAR 1000/month)	0.88	0.77; 1.01	0.073	age
**Psychological and health characteristics**				
PHQ-9 score (per standard deviation)	1.11	0.97; 1.27	0.138	disability
Stigma score (per standard deviation)	0.94	0.81; 1.08	0.353	disability
Disability score (per standard deviation)	0.99	0.86; 1.14	0.890	stigma, hypertension, ischaemic heart disease
Ischaemic heart disease	1.33	0.94; 1.89	0.104	age, depression, hypertension
Hypertension diagnosis	1.16	0.87;1.53	0.306	age, disability, ischaemic heart disease
History of TB	1.88	1.43; 2.47	**<0.001**	sex, disability, ischaemic heart diseases
Self-reported adherence ≥90%	0.35	0.24; 0.49	**<0.001**	–
ART duration <6 months or >120 months (vs 6–120 months)	2.74	1.90; 3.94	**<0.001**	age, adherence
**Outcome: Change in viral load non-suppression (HIV RNA ≥ 1000 copies/ml) from baseline to 12 months** ^**2**^				
**Socioeconomic characteristics**				
Age (per year)	0.99	0.97; 1.01	0.242	sex, hypertension, ischaemic heart disease
Female	0.95	0.56; 1.60	0.836	age
Married	0.93	0.57; 1.54	0.787	depression
Total income (per ZAR 1000/month)	0.94	0.81; 1.08	0.356	–
**Psychological and health characteristics**				
PHQ-9 score (per standard deviation)	0.92	0.75; 1.12	0.385	–
Stigma score (per standard deviation)	0.85	0.71; 1.02	0.083	–
Disability score (per standard deviation)	1.35	1.12; 1.62	**0.001**	depression, stigma, ischaemic heart disease
Ischaemic heart disease	0.88	0.57; 1.34	0.541	age, disability
Hypertension diagnosis	0.80	0.50; 1.27	0.338	age
History of TB	1.09	0.76; 1.55	0.650	sex
Self-reported adherence ≥90%	0.67	0.39; 1.16	0.149	–
ART duration <6 months or >120 months (vs 6–120 months)	0.73	0.43; 1.22	0.245	income

^1^adherence, art duration and tuberculosis also included as control variables in all models; ^2^associations with change in viral load non-suppression estimated by including viral load non-suppression at baseline as covariate in all models,; ^3^disability also included as control variable in all models

### Factors associated with body mass index

At baseline, 1854 (93%) participants had BMI and viral load values recorded and 1812 (90%) (93%) had baseline BMI values and 12 months viral load values recorded at 12 months (Table 1). Mean BMI was 26.5 (standard deviation (SD) 7.9) kg/m^2^ at baseline for those with viral load values at baseline, and 25.0 (SD 6.7) kg/m^2^ at for those with 12-month viral load values (Table 1). BMI values were available overall for 1970 (98%, mean 24.6, SD 6.7 kg/m^2^) participants at baseline, not considering the availability of viral load, and for 1562 (93%, mean 24.9, SD 6.9 kg/m^2^) of those followed up at 12 months (data not presented).

In the mixed effect linear regression model, being female, married, earning a higher income, and having hypertension were associated with greater BMI at baseline, and history of tuberculosis and viral load non-suppression were associated with lower BMI at baseline (Table 4). In the same model, BMI increased from baseline to follow-up, and older age was associated with a lesser increase from baseline to follow-up while being female was associated with a greater increase. The LASSO models showed similar associations, except that IHD was also associated with decreasing BMI from baseline to follow-up (Table 5).

**Table 4:** Association of socioeconomic, psychological and health characteristics measured at baseline with BMI at baseline and with changes in BMI from baseline to 12 months: longitudinal linear regression mixed model including all covariates.

Characteristics	BMI		
	Coefficient	95% CI	p-value
**Outcome: BMI at baseline**			
**Socioeconomic characteristics**			
Age (per year)	0.01	−0.02; 0.03	0.731
Female	4.91	4.16; 5.66	**0.001**
Married	0.76	0.09; 1.42	**0.026**
Total income (per ZAR 1000/month)	0.30	0.14; 0.46	**0.001**
**Psychological and health characteristics**			
PHQ-9 score (per standard deviation)	−0.13	−0.44; 0.17	0.395
Stigma score (per standard deviation)	0.06	−0.23; 0.34	0.694
Disability score (per standard deviation)	−0.17	−0.48; 0.14	0.277
Ischaemic heart disease	−0.05	−0.88; 0.78	0.909
Hypertension diagnosis	2.26	1.61; 2.90	**0.001**
History of TB	−1.45	−2.04; −0.85	**0.001**
Viral load non-suppression at baseline	−0.54	−0.92; −0.15	**0.007**
**Trial design**			
Intervention vs. control arm	−0.15	−1.05; 0.75	0.737
**Outcome: Change in BMI at 12 months** ^**1**^			
**Socioeconomic characteristics**			
Time from baseline to 12-months	0.80	0.13; 1.47	**0.019**
Age (per year)	−0.02	−0.04; −0.01	**0.001**
Female	0.38	0.04; 0.73	**0.029**
Married	−0.18	−0.48; 0.12	0.238
Total income (per ZAR 1000/month)	−0.002	−0.08; 0.08	0.970
**Psychological and health characteristics**			
PHQ-9 score (per standard deviation)	−0.07	−0.20; 0.07	0.346
Stigma score (per standard deviation)	−0.04	−0.17; 0.09	0.520
Disability score (per standard deviation)	0.09	−0.05; 0.23	0.198
Ischaemic heart disease	−0.35	−0.71; 0.00	0.053
Hypertension diagnosis	0.02	−0.27; 0.30	0.908
History of TB	0.07	−0.20; 0.34	0.614
Viral load non-suppression at baseline	0.23	−0.19; 0.64	0.286
**Trial design**			
Intervention vs. control arm	0.14	−0.11; 0.39	0.279

^1^Coefficients for change in outcome estimated with covariate-time interaction terms

**Table 5:** Sociodemographic and health characteristics measured at baseline associated with BMI at baseline and with change in BMI from baseline to 12 months: linear regression models with coefficients for each variable adjusted for potentially confounding covariates selected by LASSO.

Outcome and explanatory variables	BMI				
	Coefficient	95% CI		P-value	Selected control variables ^2,3^
**Outcome: BMI at baseline** ^**1**^					
Age (per year)	0.01	−0.02	0.05	0.494	ischaemic heart disease
Female	4.89	4.27	5.51	**<0.001**	age, income
Married	1.03	0.34	1.71	**0.003**	
Total income (per ZAR 1000/month)	0.29	0.11	0.47	**0.001**	age, income
PHQ-9 score (per standard deviation)	−0.21	−0.51	0.10	0.179	disability
Stigma score (per standard deviation)	0.13	−0.17	0.43	0.393	disability
Disability score (per standard deviation)	−0.21	−0.56	0.15	0.250	depression, stigma, ischaemic heart disease
Ischaemic heart disease	−0.04	−0.89	0.81	0.921	age, depression
Hypertension diagnosis	2.11	1.23	2.98	**<0.001**	age
History of TB	−1.36	−1.98	−0.74	**<0.001**	disability, ischaemic heart disease
Viral load non-suppression at baseline	−1.79	−2.42	−1.15	**<0.001**	
**Outcome: Change in BMI at 12 months** ^**2**^					
Age (per year)	−0.02	−0.03	−0.01	**<0.001**	hypertension
Female	0.65	0.25	1.06	**0.001**	age
Married	−0.14	−0.46	0.18	0.399	
Total income (per ZAR 1000/month)	0.03	−0.04	0.09	0.413	age
PHQ-9 score (per standard deviation)	−0.09	−0.25	0.06	0.239	disability
Stigma score (per standard deviation)	−0.03	−0.15	0.09	0.596	disability
Disability score (per standard deviation)	0.04	−0.07	0.15	0.509	depression, stigma, ischaemic heart disease
Ischaemic heart disease	−0.40	−0.67	−0.13	**0.004**	depression, hypertension, tuberculosis
Hypertension diagnosis	0.13	−0.21	0.48	0.452	age, ischaemic heart disease
History of TB	−0.05	−0.34	0.23	0.711	
Viral load non-suppression at baseline	0.19	−0.18	0.55	0.319	tuberculosis

^1^All models include sex, hypertension, tuberculosis and viral load non-suppression at baseline as control variables; ^2^ Associations with change in BMI estimated by including BMI at baseline as covariate in all models; ^3^sex also included as control variable in all models

## Discussion

This study showed that sociodemographic characteristics associated with viral load non-suppression among PLWH with depressive symptoms, reported in the present study, were similar to those reported in previous cross-sectional studies: younger age, being male and earning lower income [6]. These findings highlight gender and socioeconomic disparities in HIV treatment outcomes, suggesting that men and economically disadvantaged individuals may require targeted adherence support. While these associations were shown at enrolment, most were not sustained at 12 months.

The findings that both male sex and younger age are associated with viral load non-suppression is concerning. Both behavioural and structural factors may be contributing to a significantly higher risk of having viral load non-suppression in men with HIV and depression symptoms. Men tend to present to health facilities with advanced disease [47–49] and engage in alcohol consumption in the evenings which may make it more difficult to take ART [50]. There have been concerns as to whether primary care clinics in their current format are able to attract young people and men or whether alternative modes of care are required [51,52]. It is therefore important to have interventions that focus on men and young people who are on ART and at risk of depression.

The present study showed that very short or very long exposure to ART was associated with having viral load non-suppression at baseline. This underscores the importance of routine viral load monitoring and structured adherence support from the start of ART, with sustained follow-up for long-term users to prevent treatment fatigue and treatment failure. Both minimal and prolonged ART exposure were associated with decreasing likelihood of having viral load non-suppression from baseline to 12 months, potentially reflecting stability among recent initiators and treatment fatigue among newer long-term users. ART significantly reduces HIV viral load within the first few days, with many people having undetectable viral load within 1–3 months [53]. However, the reduction in viral load may be affected by adherence. Previous research has shown that people who have been on ART for longer durations may experience treatment fatigue due to pill burden and patient-provider interactions [54].

Mixed effects and LASSO models resulted in slightly different estimates of effects of very short or long ART duration on viral non-suppression at baseline and on change in viral non-suppression. The mixed effect model’s odds ratios were 8.17 and 0.30 (Table 2) and the LASSO models’ odds ratios were 2.74 and 0.73 (Table 3) respectively. These findings are, however, in keeping with the crude prevalences reported in Table 1: the percentages with viral non-suppression at baseline were 24% in participants with very long or short duration and 12% in those with intermediate duration, and at follow-up the respective percentages were 19% and 13%. In other words, viral suppression was more common in participants with very short or long ART duration both at baseline and at follow-up, but this difference was smaller at follow-up. The reason why these odds ratios were less than one when change in viral-non-suppression was the outcome was because the effect of ART duration on viral suppression at baseline had already been accounted for, and because very long or short ART duration was associated with a reduction in that difference over time. The reason why the odds ratios were closer to one in the LASSO models than in the mixed model was both because the LASSO models adjusted for fewer covariates, and because the LASSO and mixed models estimate effects on changes in different ways. Thus LASSO, as a sensitivity analysis, showed that these results were sensitive to choice of statistical method.

While self-reported ART adherence measured using VAS is subjective and prone to over-estimation, it has been shown to be strongly associated with other measures of adherence [55]. Self-reported adherence has shown reasonable correlation with viral load and other objective markers of adherence even in low-resourced settings and is more trustworthy when collected separately from clinical care [56–58]. Our finding that self-reported ART non-adherence was associated with viral load non-suppression is therefore unsurprising.

Disability was associated with increased risk of viral load non-suppression over time, suggesting that functional limitations may hinder consistent ART use. Previous studies in South Africa have shown functional disability to be associated with reduced adherence to ART [31,59]. This study further highlights its longer term role in viral load non-suppression, which is likely to be due to greater difficulty accessing and adhering to ART. Disability scores among people with depressive symptoms in this study were higher than previously reported among PLWH in South Africa [31]. Higher disability scores are an indication of functional limitations and if not addressed, may pose a risk of physical disability, lower livelihood outcomes and increased depression symptoms [32]. ART programmes, therefore, need to consider disability, mental health and rehabilitation services as part of their care package.

Although depressive symptoms at ART initiation have been associated with viral load non-suppression [60–62], in this study the severity of depressive symptoms as well as of stigma had no effect on either viral load non-suppression or BMI.

This study also confirmed the characteristics associated with higher BMI and with increasing BMI over time. Findings of higher BMI among women compared to men, and its association with hypertension are consistent with previous studies, highlighting a population at risk of this cluster of multimorbidity [20,63,64]. The rise in obesity among PLWH, especially women, may simply reflect the increasing rate of obesity in the general population. This may be influenced by South Africa’s transition to urbanization and more energy dense dietary patterns. The association of higher BMI with higher income, although in a generally impoverished population, was consistent with a previous study [65] and is likely reflective of increased access and consumption of high fat and sugar diets and access to fast foods. Cultural perceptions of a ‘healthy’ weight, where lower weight is perceived as a sign of ill-health or an indication of living with HIV, and higher weight perceived as a sign of better health, greater prosperity and being more common among people without HIV [65–68] may be contributing to obesity through excess caloric intake and consumption of high-fat diets. The association between ischaemic heart disease and decreases in BMI in the LASSO model warrants further investigation, as it may indicate unintentional weight loss due to underlying health conditions, including early stages of cardiovascular disease or other chronic illnesses.

This study had several limitations. Firstly, the high prevalence of viral load suppression in this clinic-based population constrained our statistical power to identify risk factors for viral load non-suppression. A community sample including those who have disengaged with care may have provided more insight. Secondly, we did not assess alcohol and substance misuse, which have been associated with depression, especially in men and people disengaged from care [69,70], and with virological failure [71]. This decision was informed by a low case-detection rate for alcohol misuse disorder in our pilot study in the same population [72].

Thirdly, we did not record or adjust for ART regimen although second- and third-line regimens might be associated with virological failure. However, 94% of the participants were known to be on the first-line regimen. Fourthly, as CD4 counts were no longer routinely measured, we did not assess it although it is an important indicator of immunological status and of adherence. We did not assess drug resistance, which has been reported to be at 10% in low- and middle-income countries [73]. Fifthly, although weight loss is a hallmark of HIV/AIDS, lower BMI can be an ambiguous outcome in settings such as this in which overweight and obesity are highly prevalent. Studies have also associated weight gain with ART initiation [20,64], however, our study did not explore the different regimens that could be associated with higher BMI. Sixthly, we also did not compare depressive symptoms among people receiving ART and those not living with HIV. Seventhly, the 12-month follow-up period limited our analysis of changes in outcomes. Finally, this study was conducted in South Africa, among a generally impoverished population in a country with crippling inequality [74]. A country also facing a crisis of obesity among the adult population [75]. The applicability of these findings in other contexts may differ.

The strengths of the study included the use of longitudinal data, enabling us to identify characteristics associated with viral load non-suppression and BMI at baseline and with changes during follow-up. We also used objective measures of outcomes, including our viral load data which included three sources which were triangulated. The findings of multilevel longitudinal mixed effect regression models were confirmed by the parsimonious regression models using LASSO inference methods.

### Study implications

This study shows that while short-term risk factors for viral load non-suppression include sociodemographic factors, long-term assessments must prioritize health characteristics which have a lasting impact.

Key recommendations for ART programmes include:

- Implementing supportive interventions tailored to high-risk groups like men and young people, including men and youth friendly facilities.
- Integrating comprehensive care packages that address disability, mental health, and rehabilitation services.
- Providing ongoing, long-term support beyond early treatment stages to curb treatment fatigue.
- Utilizing frequent adherence assessments (like VAS) to supplement less frequent laboratory viral load monitoring.
- Continuing weight monitoring and education on weigh to manage multimorbidity risks in PLWH.
- Future research should explore causal pathways linking disability and ART outcomes and evaluate interventions tailored to high-risk subgroups.

## Conclusions

This study found that while some sociodemographic risk factors for viral non-suppression in PLWH were consistent with previous research, many did not persist over time. Sustained risk factors included male sex, younger age, and functional disability, suggesting a need for ART programs to integrate long-term support services like frequent adherence assessment, mental health, rehabilitation, and weight management strategies tailored to high-risk groups.

## Supporting information

S1 Fig Directed acyclic graph of assumed effects of ART duration on BMI mediated through viral non-suppression. (JPG)

## References

[1] UNAIDS. Global HIV & AIDS statistics — Fact sheet 2024. https://www.unaids.org/en/resources/fact-sheet#:~:text=And%20among%20people%20accessing%20treatment,domestic%20HIV%20budgets%20by%202026. Accessed 2025 November 25.

[2] UNAIDS. UNAIDS AIDS info country factsheets South Africa 2015. UNAIDS. 2022.

[3] McLeod W, Bor J, Crawford K, Carmona S. Analysis of Big Data for better targeting of ART Adherence Strategies: Spatial clustering analysis of viral load suppression by South African province, district, sub-district and facility (April 2014–March 2015). In: National Department of Health, editor. Johannesburg, South Africa: National Department of Health. 2015.

[4] Joseph Davey D, Abrahams Z, Feinberg M, Prins M, Serrao C, Medeossi B, et al. Factors associated with recent unsuppressed viral load in HIV-1-infected patients in care on first-line antiretroviral therapy in South Africa. Int J STD AIDS. 2018;29(6):603–10. doi: 10.1177/0956462417748859 29334886 PMC7053422

[5] Sunpath H, Hatlen TJ, Naidu KK, Msimango P, Adams RN, Moosa M-YS, et al. Targeting the third “90”: introducing the viral load champion. Public Health Action. 2018;8(4):225–31. doi: 10.5588/pha.18.0063 30775284 PMC6361489

[6] Mosha IH, Nyondo GG, Munishi CG, Njiro BJ, Bwire GM. Prevalence and factors associated with viral non-suppression in people living with HIV receiving antiretroviral therapy in sub-Saharan Africa: A systematic review and meta-analysis. Rev Med Virol. 2024;34(3):e2540. doi: 10.1002/rmv.2540 38708846 PMC11829566

[7] Safari F, Hashempour A, Khodadad N, Alinazari MMK, Akbarinia S, Tabatabaee SA. What leads to treatment failure among people living with HIV: first systematic review from the middle east and North Africa. Virol J. 2025;22(1):344. doi: 10.1186/s12985-025-02916-2 41146253 PMC12560295

[8] Huang B, Younger A, Gallant MP, O’Grady TJ. Depressive Symptoms and HIV Viral Suppression: A Systematic Review and Meta-analysis. AIDS Behav. 2025;29(3):870–83. doi: 10.1007/s10461-024-04571-0 39690344 PMC11830644

[9] van Liere GAFS, Lilian R, Dunlop J, Tait C, Rees K, Mabitsi M, et al. High rate of loss to follow-up and virological non-suppression in HIV-infected children on antiretroviral therapy highlights the need to improve quality of care in South Africa. Epidemiol Infect. 2021;149:e88. doi: 10.1017/S0950268821000637 33745490 PMC8080219

[10] Tomescu S, Crompton T, Adebayo J, Akpan F, Dauda DS, Allen Z, et al. Factors associated with viral load non-suppression in people living with HIV on ART in Nigeria: cross-sectional analysis from 2017 to 2021. BMJ Open. 2023;13(5):e065950. doi: 10.1136/bmjopen-2022-065950 37169497 PMC10186439

[11] Munyayi FK, van Wyk B. Closing the HIV Treatment Gap for Adolescents in Windhoek, Namibia: A Retrospective Analysis of Predictors of Viral Non-Suppression. Int J Environ Res Public Health. 2022;19(22):14710. doi: 10.3390/ijerph192214710 36429431 PMC9690371

[12] Merrill KG, Campbell JC, Decker MR, McGready J, Burke VM, Mwansa JK, et al. Past-Year Violence Victimization is Associated with Viral Load Failure Among HIV-Positive Adolescents and Young Adults. AIDS Behav. 2021;25(5):1373–83. doi: 10.1007/s10461-020-02958-3 32761474 PMC8052241

[13] Bitwale NZ, Mnzava DP, Kimaro FD, Jacob T, Mpondo BCT, Jumanne S. Prevalence and Factors Associated With Virological Treatment Failure Among Children and Adolescents on Antiretroviral Therapy Attending HIV/AIDS Care and Treatment Clinics in Dodoma Municipality, Central Tanzania. J Pediatric Infect Dis Soc. 2021;10(2):131–40. doi: 10.1093/jpids/piaa030 32463083

[14] Afrane AKA, Goka BQ, Renner L, Yawson AE, Alhassan Y, Owiafe SN, et al. HIV virological non-suppression and its associated factors in children on antiretroviral therapy at a major treatment centre in Southern Ghana: a cross-sectional study. BMC Infect Dis. 2021;21(1):731. doi: 10.1186/s12879-021-06459-z 34340689 PMC8330060

[15] Bailin SS, Koethe JR, Rebeiro PF. The pathogenesis of obesity in people living with HIV. Curr Opin HIV AIDS. 2024;19(1):6–13. doi: 10.1097/COH.0000000000000834 37934696 PMC10842175

[16] World Health Organization. Obesity and overweight. https://www.who.int/news-room/fact-sheets/detail/obesity-and-overweight. 2021.

[17] Millman N, Koethe JR, Erlandson KM. Obesity among women with HIV. Curr Opin HIV AIDS. 2024;19(1):30–4. doi: 10.1097/COH.0000000000000828 37909915 PMC10842230

[18] Hernandez D, Kalichman S, Cherry C, Kalichman M, Washington C, Grebler T. Dietary intake and overweight and obesity among persons living with HIV in Atlanta Georgia. AIDS Care. 2017;29(6):767–71. doi: 10.1080/09540121.2016.1238441 27723990

[19] Vancampfort D, Mugisha J, De Hert M, Probst M, Firth J, Gorczynski P, et al. Global physical activity levels among people living with HIV: a systematic review and meta-analysis. Disabil Rehabil. 2018;40(4):388–97. doi: 10.1080/09638288.2016.1260645 27929355

[20] Bares SH, Smeaton LM, Xu A, Godfrey C, McComsey GA. HIV-Infected Women Gain More Weight than HIV-Infected Men Following the Initiation of Antiretroviral Therapy. Journal of Women’s Health (2002). 2018;27(9):1162–9. 10.1089/jwh.2017.6717 PMC6148723 29608129

[21] Bakal DR, Coelho LE, Luz PM, Clark JL, De Boni RB, Cardoso SW, et al. Obesity following ART initiation is common and influenced by both traditional and HIV-/ART-specific risk factors. J Antimicrob Chemother. 2018;73(8):2177–85. doi: 10.1093/jac/dky145 29722811 PMC6054231

[22] Hasse B, Iff M, Ledergerber B, Calmy A, Schmid P, Hauser C, et al. Obesity Trends and Body Mass Index Changes After Starting Antiretroviral Treatment: The Swiss HIV Cohort Study. Open Forum Infect Dis. 2014;1(2):ofu040. doi: 10.1093/ofid/ofu040 25734114 PMC4281814

[23] Ngandu NK, Lombard CJ, Mbira TE, Puren A, Waitt C, Prendergast AJ, et al. HIV viral load non-suppression and associated factors among pregnant and postpartum women in rural northeastern South Africa: a cross-sectional survey. BMJ Open. 2022;12(3):e058347. doi: 10.1136/bmjopen-2021-058347 35273061 PMC8915310

[24] Zani B, Fairall L, Petersen I, Folb N, Bhana A, Hanass-Hancock J, et al. Effectiveness of a task-sharing collaborative care model for the detection and management of depression among adults receiving antiretroviral therapy in primary care facilities in South Africa: A pragmatic cluster randomised controlled trial. J Affect Disord. 2025;370:499–510. doi: 10.1016/j.jad.2024.10.061 39442695

[25] Fairall L, Petersen I, Zani B, Folb N, Georgeu-Pepper D, Selohilwe O, et al. Collaborative care for the detection and management of depression among adults receiving antiretroviral therapy in South Africa: study protocol for the CobALT randomised controlled trial. Trials. 2018;19(1):193. doi: 10.1186/s13063-018-2517-7 29566739 PMC5863840

[26] Bhana A, Rathod SD, Selohilwe O, Kathree T, Petersen I. The validity of the Patient Health Questionnaire for screening depression in chronic care patients in primary health care in South Africa. BMC Psychiatry. 2015;15:118. doi: 10.1186/s12888-015-0503-0 26001915 PMC4446842

[27] Kroenke K, Spitzer RL, Williams JB. The PHQ-9: validity of a brief depression severity measure. J Gen Intern Med. 2001;16(9):606–13. doi: 10.1046/j.1525-1497.2001.016009606.x 11556941 PMC1495268

[28] Kalichman SC, Simbayi LC, Cloete A, Mthembu PP, Mkhonta RN, Ginindza T. Measuring AIDS stigmas in people living with HIV/AIDS: the Internalized AIDS-Related Stigma Scale. AIDS Care. 2009;21(1):87–93. doi: 10.1080/09540120802032627 19085224

[29] Cohen S, Kamarck T, Mermelstein R. A global measure of perceived stress. J Health Soc Behav. 1983;24(4):385–96. doi: 10.2307/2136404 6668417

[30] Garin O, Ayuso-Mateos JL, Almansa J, Nieto M, Chatterji S, Vilagut G, et al. Validation of the “World Health Organization Disability Assessment Schedule, WHODAS-2” in patients with chronic diseases. Health Qual Life Outcomes. 2010;8:51. doi: 10.1186/1477-7525-8-51 20482853 PMC2893517

[31] Hanass-Hancock J, Myezwa H, Carpenter B. Disability and Living with HIV: Baseline from a Cohort of People on Long Term ART in South Africa. PLoS One. 2015;10(12):e0143936. doi: 10.1371/journal.pone.0143936 26625001 PMC4666651

[32] Myezwa H, Hanass-Hancock J, Ajidahun AT, Carpenter B. Disability and health outcomes - from a cohort of people on long-term anti-retroviral therapy. SAHARA J. 2018;15(1):50–9. doi: 10.1080/17290376.2018.1459813 29635976 PMC5917329

[33] Ustun T, Kostanjesek N, Chatterji S, Rehm J, World Health Organization. Measuring health and disability: manual for WHO Disability Assessment Schedule (WHODAS 2.0). Geneva, Switzerland: World Health Organization. 2010.

[34] Amico KR, Fisher WA, Cornman DH, Shuper PA, Redding CG, Konkle-Parker DJ, et al. Visual analog scale of ART adherence: association with 3-day self-report and adherence barriers. J Acquir Immune Defic Syndr. 2006;42(4):455–9. doi: 10.1097/01.qai.0000225020.73760.c2 16810111

[35] Anderson K, Muloiwa R, Davies M-A. Treatment outcomes in perinatally infected HIV-positive adolescents and young adults after ≥10 years on antiretroviral therapy. S Afr Med J. 2018;109(1):27–34. doi: 10.7196/SAMJ.2018.v109i1.13230 30606301

[36] Joint United Nations Programme on HIV/AIDS U. Global AIDS response progress reporting 2016: Construction of core indicators for monitoring the 2011 United Nations Political Declaration on HIV and AIDS. Geneva, Switzerland: UNAIDS. 2016.

[37] Froot KA. Consistent Covariance Matrix Estimation with Cross-Sectional Dependence and Heteroskedasticity in Financial Data. The Journal of Financial and Quantitative Analysis. 1989;24(3):333. doi: 10.2307/2330815

[38] Williams RL. A note on robust variance estimation for cluster-correlated data. Biometrics. 2000;56(2):645–6. doi: 10.1111/j.0006-341x.2000.00645.x 10877330

[39] Austin PC. Using the Standardized Difference to Compare the Prevalence of a Binary Variable Between Two Groups in Observational Research. Communications in Statistics - Simulation and Computation. 2009;38(6):1228–34. doi: 10.1080/03610910902859574

[40] Verbeke G, Molenberghs G. Linear mixed models for longitudinal data. 2000th ed. New York: Springer. 2000.

[41] Belloni A, Chernozhukov V, Wei Y. Post-Selection Inference for Generalized Linear Models With Many Controls. Journal of Business & Economic Statistics. 2016;34(4):606–19. doi: 10.1080/07350015.2016.1166116

[42] Belloni A, Chernozhukov V, Hansen C. Inference on Treatment Effects after Selection among High-Dimensional Controls. The Review of Economic Studies. 2013;81(2):608–50. doi: 10.1093/restud/rdt044

[43] StataCorp. Stata 19 Lasso Reference Manual. College Station, Texas: Stata Press. 2025.

[44] Vickers AJ, Altman DG. Statistics notes: Analysing controlled trials with baseline and follow up measurements. BMJ. 2001;323(7321):1123–4. doi: 10.1136/bmj.323.7321.1123 11701584 PMC1121605

[45] Stata Statistical Software: Release 13. http://www.stata.com. Accessed 2023 October 1.

[46] Nedbank Limited South Africa. Annual average exchange rates 2016. Nedbank Limited South Africa. 2016. https://www.nedbank.co.za/content/dam/nedbank/site-assets/AboutUs/Economics_Unit/Forecast_and_data/Daily_Rates/Annual_Average_Exchange_Rates.pdf

[47] South African National AIDS Council. South Africa’s National Strategic Plan for HIV, TB and STIs 2017-2022. Pretoria: Health Department. 2017.

[48] Carmona S, Bor J, Nattey C, Maughan-Brown B, Maskew M, Fox MP, et al. Persistent High Burden of Advanced HIV Disease Among Patients Seeking Care in South Africa’s National HIV Program: Data From a Nationwide Laboratory Cohort. Clin Infect Dis. 2018;66(suppl_2):S111–7. doi: 10.1093/cid/ciy045 29514238 PMC5850436

[49] Cornell M, Schomaker M, Garone DB, Giddy J, Hoffmann CJ, Lessells R, et al. Gender differences in survival among adult patients starting antiretroviral therapy in South Africa: a multicentre cohort study. PLoS Med. 2012;9(9):e1001304. doi: 10.1371/journal.pmed.1001304 22973181 PMC3433409

[50] Hanass-Hancock J, Carpenter B, Myezwa H. The missing link: exploring the intersection of gender, capabilities, and depressive symptoms in the context of chronic HIV. Women Health. 2019;59(10):1212–26. doi: 10.1080/03630242.2019.1607799 31043146

[51] Cornell M. Gender inequality: Bad for men’s health. South Afr J HIV Med. 2013;14(1):12–4. doi: 10.7196/SAJHIVMED.894 24078805 PMC3782744

[52] Cornell M, Cox V, Wilkinson L. Public health blindness towards men in HIV programmes in Africa. Trop Med Int Health. 2015;20(12):1634–5. doi: 10.1111/tmi.12593 26325025

[53] Hoenigl M, Chaillon A, Moore DJ, Morris SR, Mehta SR, Gianella S, et al. Rapid HIV Viral Load Suppression in those Initiating Antiretroviral Therapy at First Visit after HIV Diagnosis. Sci Rep. 2016;6:32947. doi: 10.1038/srep32947 27597312 PMC5011704

[54] Claborn KR, Meier E, Miller MB, Leffingwell TR. A systematic review of treatment fatigue among HIV-infected patients prescribed antiretroviral therapy. Psychol Health Med. 2015;20(3):255–65. doi: 10.1080/13548506.2014.945601 25110152 PMC4315727

[55] Finitsis DJ, Pellowski JA, Huedo-Medina TB, Fox MC, Kalichman SC. Visual analogue scale (VAS) measurement of antiretroviral adherence in people living with HIV (PLWH): a meta-analysis. J Behav Med. 2016;39(6):1043–55. doi: 10.1007/s10865-016-9770-6 27481102

[56] Nieuwkerk PT, Oort FJ. Self-reported adherence to antiretroviral therapy for HIV-1 infection and virologic treatment response: a meta-analysis. J Acquir Immune Defic Syndr. 2005;38(4):445–8. doi: 10.1097/01.qai.0000147522.34369.12 15764962

[57] Mekuria LA, Prins JM, Yalew AW, Sprangers MAG, Nieuwkerk PT. Which adherence measure - self-report, clinician recorded or pharmacy refill - is best able to predict detectable viral load in a public ART programme without routine plasma viral load monitoring? Trop Med Int Health. 2016;21(7):856–69. doi: 10.1111/tmi.12709 27118068

[58] Phillips TK, Wilson IB, Brittain K, Zerbe A, Mellins CA, Remien RH, et al. Decreases in Self-Reported ART Adherence Predict HIV Viremia Among Pregnant and Postpartum South African Women. J Acquir Immune Defic Syndr. 2019;80(3):247–54. doi: 10.1097/QAI.0000000000001909 30422906 PMC6375758

[59] Carpenter BS, Hanass-Hancock J, Myezwa H. Looking at antiretroviral adherence through a disability lens: a cross-sectional analysis of the intersection of disability, adherence, and health status. Disabil Rehabil. 2020;42(6):806–13. doi: 10.1080/09638288.2018.1510048 30616436

[60] Bengtson AM, Pence BW, Mimiaga MJ, Gaynes BN, Moore R, Christopoulos K, et al. Depressive Symptoms and Engagement in Human Immunodeficiency Virus Care Following Antiretroviral Therapy Initiation. Clin Infect Dis. 2019;68(3):475–81. doi: 10.1093/cid/ciy496 29901695 PMC6336906

[61] Ironson G, O’Cleirigh C, Fletcher MA, Laurenceau JP, Balbin E, Klimas N, et al. Psychosocial factors predict CD4 and viral load change in men and women with human immunodeficiency virus in the era of highly active antiretroviral treatment. Psychosom Med. 2005;67(6):1013–21. doi: 10.1097/01.psy.0000188569.58998.c8 16314608 PMC2614887

[62] Ickovics JR, Hamburger ME, Vlahov D, Schoenbaum EE, Schuman P, Boland RJ. Mortality, CD4 cell count decline, and depressive symptoms among HIV-seropositive women: longitudinal analysis from the HIV Epidemiology Research Study. JAMA. 2001;285(11):1466–74. 11255423 10.1001/jama.285.11.1466

[63] Lartey M, Torpey K, Ganu V, Ayisi Addo S, Bandoh D, Abdulai M, et al. Hypertension Among Cohort of Persons With Human Immunodeficiency Virus Initiated on a Dolutegravir-Based Antiretroviral Regimen in Ghana. Open Forum Infect Dis. 2024;11(3):ofae061. doi: 10.1093/ofid/ofae061 38444823 PMC10913832

[64] Cardoso-Neto ÉC, Netto EM, Brites C. Weight gain in patients starting Dolutegravir-based ART according to baseline CD4 count after 48 weeks of follow up. Braz J Infect Dis. 2023;27(5):102807. doi: 10.1016/j.bjid.2023.102807 37788801 PMC10569987

[65] Becker N, Mkhonta A, Sibeko LN. The prevalence of overweight/obesity and its association with household food insecurity among women living with HIV in rural Eswatini. BMC Public Health. 2022;22(1):629. doi: 10.1186/s12889-022-13036-9 35361183 PMC8969360

[66] Manne-Goehler J, Rahim N, van Empel E, de Vlieg R, Chamberlin G, Ihama A, et al. Perceptions of Health, Body Size, and Nutritional Risk Factors for Obesity in People with HIV in South Africa. AIDS Behav. 2024;28(1):367–75. doi: 10.1007/s10461-023-04152-7 37632604 PMC10841992

[67] Hyle EP, Martey EB, Bekker L-G, Xu A, Parker RA, Walensky RP, et al. Diet, physical activity, and obesity among ART-experienced people with HIV in South Africa. AIDS Care. 2023;35(1):71–7. doi: 10.1080/09540121.2021.2012556 34913762 PMC9200895

[68] Hanley S, Moodley D, Naidoo M. Obesity in young South African women living with HIV: A cross-sectional analysis of risk factors for cardiovascular disease. PLoS One. 2021;16(11):e0255652. doi: 10.1371/journal.pone.0255652 34780476 PMC8592426

[69] Cavanagh A, Wilson CJ, Caputi P, Kavanagh DJ. Symptom endorsement in men versus women with a diagnosis of depression: A differential item functioning approach. Int J Soc Psychiatry. 2016;62(6):549–59. doi: 10.1177/0020764016653980 27335340

[70] Cichowitz C, Maraba N, Hamilton R, Charalambous S, Hoffmann CJ. Depression and alcohol use disorder at antiretroviral therapy initiation led to disengagement from care in South Africa. PLoS One. 2017;12(12):e0189820. doi: 10.1371/journal.pone.0189820 29281681 PMC5744960

[71] Nakimuli-Mpungu E, Bass JK, Alexandre P, Mills EJ, Musisi S, Ram M, et al. Depression, alcohol use and adherence to antiretroviral therapy in sub-Saharan Africa: a systematic review. AIDS Behav. 2012;16(8):2101–18. doi: 10.1007/s10461-011-0087-8 22116638

[72] Petersen I, Bhana A, Fairall LR, Selohilwe O, Kathree T, Baron EC, et al. Evaluation of a collaborative care model for integrated primary care of common mental disorders comorbid with chronic conditions in South Africa. BMC Psychiatry. 2019;19(1):107. doi: 10.1186/s12888-019-2081-z 30943947 PMC6448306

[73] Gupta RK, Gregson J, Parkin N, Haile-Selassie H, Tanuri A, Andrade Forero L, et al. HIV-1 drug resistance before initiation or re-initiation of first-line antiretroviral therapy in low-income and middle-income countries: a systematic review and meta-regression analysis. Lancet Infect Dis. 2018;18(3):346–55. doi: 10.1016/S1473-3099(17)30702-8 29198909 PMC5835664

[74] World Bank. Inequality in Southern Africa: An Assessment of the Southern African Customs Union. Washington, DC: World Bank. 2022.

[75] Govender I, Sunnasy A. The growing problem of obesity in South Africa. S Afr Fam Pract (2004). 2025;67(1):e1–2. doi: 10.4102/safp.v67i1.6001 39935151 PMC11830878

