# Supplementary figures and images for: Sociodemographic and health-related factors associated with viral load non-suppression and body mass index in adults with depression symptoms receiving antiretroviral therapy in South Africa
Source: PLoS One. 2026 Feb 23;21(2):e0329990. doi: 10.1371/journal.pone.0329990 (PMC12928420; doi:10.1371/journal.pone.0329990)

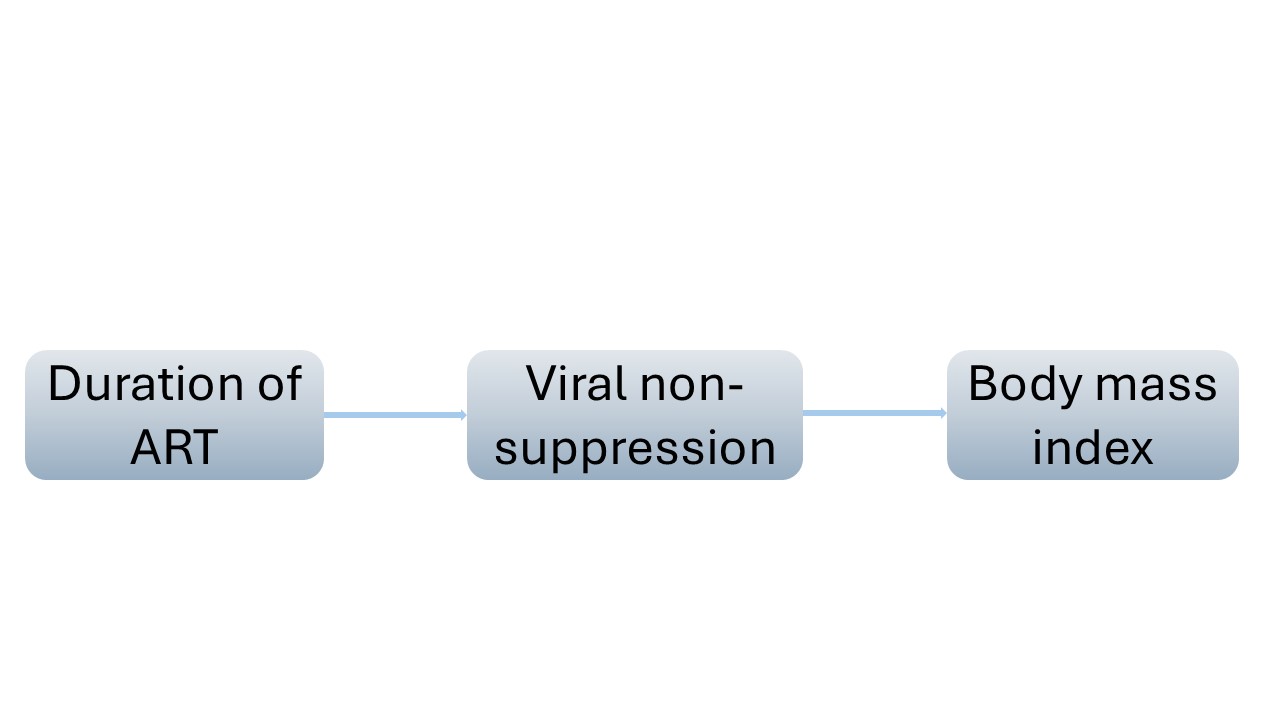

Supplement: S1 Fig — (JPG) [file pone.0329990.s001.jpg]
